# Errata

**DOI:** 10.36660/abc.20240681

**Published:** 2024-10-24

Arq Bras Cardiol. 2024; 121(7):e20240478

Na “Diretriz Brasileira sobre a Saúde Cardiovascular no Climatério e na Menopausa – 2024”, com número de DOI: https://doi.org/10.36660/abc.20240478, publicado no periódico Arquivos Brasileiros de Cardiologia, Arq Bras Cardiol. 2024; 121(7):e20240478, na página 24, Figura 4.1, no quadro verde “RISCO BAIXO”, corrigir “METAS: LDL-c < 100 mg/dL, não HDL-c < 130 mg/dL” por “METAS: LDL-c < 130 mg/dL, não HDL-c < 160 mg/dL”. Ver figura corrigida abaixo:

**Figure:**
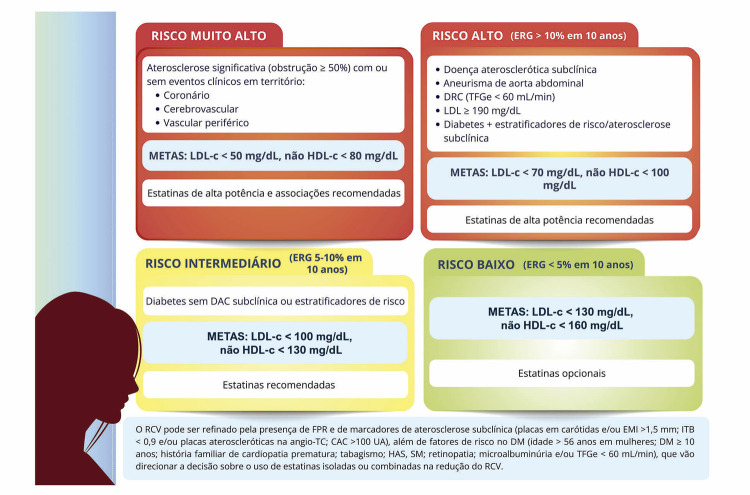

Arq Bras Cardiol. 2024; 121(8):e20240525

Na “Diretriz Brasileira de Ergometria em Crianças e Adolescentes – 2024”, com número de DOI: https://doi.org/10.36660/abc.20240525, publicado no periódico Arquivos Brasileiros de Cardiologia, Arq Bras Cardiol. 2024; 121(8):e20240525, na página 1, foi incluído, na autoria, nome e instituições, conforme abaixo. E, na página 3, acrescentamos o conflito de interesse da autora: Nada a ser declarado.

Ana Luíza Guimarães Ferreira^22,23^

https://orcid.org/0000-0003-4535-4884

Instituto Dante Pazzanese de Cardiologia,^22^ São Paulo, SP – Brasil

Hospital Israelita Albert Einstein,^23^ São Paulo, SP – Brasil

