# Supplementary material for: Errata
Source: Arq Bras Cardiol. 2024 Oct 24;121(10):e20240681. [Article in Portuguese] doi: 10.36660/abc.20240681 (PMC11649000; doi:10.36660/abc.20240681)
Supplement: Translation

# Translation: Erratum

**DOI:** 10.36660/abc.20240681i

Arq Bras Cardiol. 2024; 121(7):e20240478

In the “Brazilian Guideline on Menopausal Cardiovascular Health – 2024,” with DOI number: https://doi.org/10.36660/abc.20240478i, published in the journal Arquivos Brasileiros de Cardiologia, Arq Bras Cardiol. 2024; 121(7):e20240478, on page 23, Figure 4.1, in the green box “LOW RISK,” please correct “GOALS: LDL-c < 100 mg/dL, non-HDL-c < 130 mg/dL” to “GOALS: LDL-c < 130 mg/dL, non-HDL-c < 160 mg/dL.”

Arq Bras Cardiol. 2024; 121(8):e20240525

In the “Brazilian Guideline for Exercise Testing in Children and Adolescents – 2024,” with DOI number: https://doi.org/10.36660/abc.20240525i, published in the journal Arquivos Brasileiros de Cardiologia, Arq Bras Cardiol. 2024; 121(8):e20240525, on page 1, the author’s name and institutions were included as below. Additionally, on page 3, we added the author’s conflict of interest: Nothing to be declare.

Ana Luíza Guimarães Ferreira^22,23^

https://orcid.org/0000-0003-4535-4884

Instituto Dante Pazzanese de Cardiologia,^22^ São Paulo, SP – Brazil

Hospital Israelita Albert Einstein,^23^ São Paulo, SP – Brazil
